# Association between adenomyosis subtypes and concurrent endometrial lesions: a propensity score-matched retrospective study

**DOI:** 10.3389/fendo.2026.1842535

**Published:** 2026-05-29

**Authors:** Xiaoxi Niu, Yiyi Wang, Yijie Zhai, Yunyan Teng, Zhaogang Dong, Lijie Wang

**Affiliations:** 1 Department of Obstetrics and Gynecology, Qilu Hospital of Shandong University, Jinan, Shandong, China; 2 Department of Clinical Laboratory, Qilu Hospital of Shandong University, Jinan, Shandong, China

**Keywords:** adenomyosis, endometrial lesions, phenotypic heterogeneity, propensity score matching, reproductive health

## Abstract

**Background:**

Adenomyosis is increasingly recognized as a heterogeneous syndrome comprising focal and diffuse subtypes. While adenomyosis is known to be associated with endometrial lesions, it remains unclear whether this risk varies between specific phenotypes. This study aimed to evaluate the association of the diffuse adenomyosis phenotype with the risk of co-existing endometrial lesions using propensity score matching (PSM).

**Methods:**

A retrospective study was conducted on 685 patients with confirmed adenomyosis between January 2018 and December 2023. Patients were classified into focal (Fo-ADS, n=404) and diffuse (Di-ADS, n=281) subtypes. To minimize selection bias from baseline confounders, a 1:1 PSM was performed based on age, body mass index (BMI), parity, pain severity, CA125 levels, and history of endometriosis. Multivariate conditional logistic regression and subgroup analyses were employed to determine the correlated risk of endometrial lesions.

**Results:**

Prior to matching, Di-ADS patients were significantly older, had a higher BMI, greater parity, and more severe pain, but exhibited a lower prevalence of coexisting endometriosis compared to the Fo-ADS group. The overall incidence of endometrial lesions was significantly higher in the Di-ADS group (49.5% vs. 35.6%, P<0.001). After successfully matching 188 patient pairs (n=376), all baseline covariates were optimally balanced. Conditional logistic regression demonstrated that diffuse adenomyosis remained a significant risk factor for concurrent endometrial lesions (adjusted odds ratio [aOR] = 2.05, 95% CI: 1.28~3.27, P = 0.003). Subgroup analysis revealed a marginal interaction for age (P for interaction = 0.058), with a potentially stronger association observed in woman aged ≤45 years (P <.001).

**Conclusions:**

Focal and diffuse adenomyosis represent distinct clinical phenotypes. Diffuse adenomyosis is associated with an increased risk of endometrial lesions, irrespective of age and metabolic factors. Vigilant endometrial surveillance is strongly mandated for patients with diffuse adenomyosis, particularly in women aged 45 years or younger.

## Introduction

Adenomyosis is a uterine disorder characterized by invasion of endometrial tissue into the myometrium, typically accompanied by surrounding myometrial cell hypertrophy and hyperplasia (1), resulting in myometrial thickening and uterine enlargement (2). Clinically, the main symptoms of adenomyosis are menorrhagia, dysmenorrhea and infertility, which profoundly impair quality of life and affect tens to hundreds of millions of reproductive-age women (3). Although its estimated prevalence ranging from approximately 1% in the general population to 20-49% among symptomatic women (4), the etiology and pathogenesis remain incompletely understood (5). Emerging evidence indicates that adenomyosis is a heterogeneous syndrome composed of distinct phenotypes, rather than a single uniform disease entity (6). Its pathogenesis involves disruption of the endometrial-myometrial interface (EMI), repeated tissue injury and repair (TIAR), hormonal dysregulation, and chronic inflammation (7, 8).

Based on the extent of myometrial involvement, adenomyosis is classified as diffuse or focal subtypes that differ not only in imaging appearance but also in underlying pathophysiology. The diffuse adenomyosis involves widespread thickening of the junctional zone throughout the myometrium, whereas focal type presents as localized, circumscribed lesions (5). Recent large-scale studies have demonstrated that diffuse adenomyosis is often considered a progressive phenotype associated with the TIAR theory. In contrast, focal adenomyosis shows links with deep infiltrating endometriosis (DIE) related to Müllerian metaplasia or extrinsic invasion, particularly when located in the posterior wall (9).

Endometrial lesions are frequently observed in women with adenomyosis (10). This association is biologically plausible because adenomyosis is estrogen-dependent and characterized by a locally progesterone resistance (11). Moreover, hormonal imbalance together with chronic inflammation and impaired decidualization can create a microenvironment that favors concurrent endometrial pathology (12). Prior hysterectomy-based studies have demonstrated that adenomyosis is associated with endometrial polyps and cancer (13), but these analyses did not stratify by adenomyosis phenotype, leaving unresolved whether the risk is uniform across disease or driven by specific phenotypes. Given that diffuse adenomyosis entails more extensive hormonal and inflammatory dysregulation, we hypothesized that diffuse adenomyosis would be related to a higher risk of endometrial lesions than focal adenomyosis.

To test this hypothesis, we conducted a retrospective study of 685 patients with confirmed adenomyosis and applied propensity score matching (PSM) to isolate the association between the diffuse phenotype and endometrial lesions and thereby provide insights to inform more personalized clinical management.

## Materials and methods

### Study design and population

This retrospective study was conducted at the Department of Obstetrics and Gynecology, Qilu Hospital of Shandong University. We reviewed the medical records of patients diagnosed with adenomyosis between January 2018 and December 2023. A total of 685 women were enrolled. The study protocol was approved by the Institutional Review Board of Qilu Hospital of Shandong University and complied with the Declaration of Helsinki.

Patients could be enrolled if they met the following criteria (1): pathologically confirmed diagnosis of adenomyosis based on hysterectomy specimens or adenomyoma excision specimens; (2) Clinically/Sonographically Diagnosed Cases: Patients who underwent hysteroscopy; (3) pathological confirmation of endometrial assessment; (4) age≥18 years at the time of surgery; and (5) complete medical records including preoperative clinical data, intraoperative findings, and postoperative pathological reports. Exclusion criteria included current pregnancy or lactation, history of previous surgery for adenomyosis and incomplete clinical or pathological data.

### Data extraction

Clinical and demographic characteristics were comprehensively extracted from electronic medical records. Age was stratified into <35, 35-44, 45-54, and≥55 years. Body Mass Index (BMI) was calculated and categorized according to the Working Group on Obesity in China (WGOC) criteria: underweight (<18.5 kg/m^2^), normal (18.5-23.9 kg/m^2^), overweight (24-27.9 kg/m^2^), and obesity (≥28 kg/m^2^). Obstetric history included times of gravidity, parity and abortion. Severity of dysmenorrhea and pelvic pain was assessed using the Visual Analogue Scale (VAS), categorized as none/mild (VAS 0-3), moderate (VAS 4-6), or severe (VAS 7-10). Menorrhagia was defined as menstrual blood loss exceeding 80 mL per cycle or periods lasting more than 7 days. A menstrual cycle length <21 days or >35 days was defined as an irregular menstrual cycle. Preoperative hematological parameters obtained within 7 days prior to surgery, including blood routine, coagulation indices and carbohydrate antigen 125 (CA125). The Systemic Immune-Inflammation Index (SII) was calculated as follows: SII = (platelet count × neutrophil count)/lymphocyte count.

Routine preoperative pelvic ultrasonography by transvaginal or transabdominal using Morphological Uterus Sonographic Assessment (MUSA) definitions was performed. Magnetic resonance imaging (MRI), surgical details, and pathological diagnoses were also recorded. Morphological features included uterine length (L; from the fundus to the internal os), transverse (T) diameter, and anteroposterior (AP) diameter (mm). The uterus size was calculated by the formula: Volume=L ×T × AP×π/6 (14). Adenomyosis was diagnosed pathologically by the presence of endometrial glands ≥3 mm from the endometrial–myometrial junction. Preoperative and postoperative findings, including leiomyomas, endometriosis, and other gynecologic conditions, were also recorded.

### Definition of adenomyosis subtypes and endometrial lesion

Adenomyosis was classified into focal adenomyosis (Fo-ADS) and diffuse adenomyosis (Di-ADS) subtypes based on imaging and pathology. An adenomyotic lesion was defined as focal if > 25% of its circumference was surrounded by normal myometrium including adenomyoma, while diffuse adenomyosis was defined as adenomyosis patterns extending throughout the myometrium or with <25% of the lesion circumference surrounded by normal myometrium (15, 16). In cases where both patterns coexisted, classification was based on the predominant phenotype in our study.

Endometrial lesions were defined as the presence of any of the following pathologically confirmed conditions: (1) endometrial polyps; (2) endometrial hyperplasia without atypia; (3) atypical endometrial hyperplasia (endometrial intraepithelial neoplasia); or (4) endometrial cancer (17). The composite outcome of “endometrial lesion” included all four categories. All pathological diagnoses were reviewed by two experienced gynecological pathologists, and discrepancies were resolved by consensus.

### Statistical analysis

Continuous variables were evaluated for normality. Data following a normal distribution are reported as mean ± standard deviation (SD) and were compared using an independent-samples t-test. Non-normally distributed variables were presented as median with interquartile range (IQR, Q1-Q3) and compared with the Mann-Whitney U test. Categorical variables are shown as frequencies and percentages (n, %) and were assessed by the chi-square test or Fisher’s exact test, as appropriate.

To minimize selection bias from baseline differences between the focal and diffuse groups, PSM was performed. Propensity scores were calculated, and a 1:1 nearest-neighbor matching algorithm with a caliper width of 0.02 was applied. Covariant balance before and after matching was evaluated using the standardized mean difference (SMD), with SMD<0.1 indicating acceptable balance. Finally, a multivariable binary logistic regression was conducted to identify risk factors. To evaluate the consistency of the association between adenomyosis subtype and endometrial lesions, subgroup analyses was performed. For subgroup analyses, multivariable adjusted odds ratios (ORs) and 95% confidence intervals (CIs) were calculated. Covariates included age, BMI, parity, and hormone therapy. To avoid over-adjustment, the stratifying variable itself was excluded from the multivariable model within its corresponding subgroup analysis. Interaction terms were introduced to calculate the P for interaction across different strata.

Data processing and statistical evaluations were conducted through SPSS (v25.0; IBM Corp., Armonk, NY) and R (v4.2.0; R Foundation for Statistical Computing, Vienna). For all tests, statistical significance was predefined as a two-tailed P < 0.05. Scale bars were added to the micrographs using ImageJ software (version 1.54p; National Institutes of Health, USA).

## Results

A total of 685 patients diagnosed with adenomyosis were included in this study (**Table 1**). Among them, 404 patients (59.0%) were classified as Fo-ADS, while 281 cases (41.0%) as Di-ADS. The mean age at surgery was 44.03 ± 6.6years old (range: 25–65 years), with the majority of patients aged 35–44 years (42.3%) or 45–54 years (43.8%), and only 2.5% were more than 55 years. Overall, 59.3% of participants were classified as overweight or obese (BMI ≥ 24 kg/m^2^) according to WGOC criteria. Patients in Di-ADS group were significantly older (45.33 ± 6.51 vs. 43.13 ± 6.55 years, P<0.001) and had a higher BMI (25.62± 3.82 vs. 24.81 ± 3.62 kg/m^2^, P = 0.005) compared with those in Fo-ADS group (**Table 2**). Analysis of obstetric history revealed that 4.2% of patients were nulligravid and 8.6% were nulliparous, while the majority (86.9%) had a parity of 1 to 2 times. Parity was significantly higher in the Di-ADS group (1.41 ± 0.74 vs. 1.28 ± 0.73, P = 0.011). A history of abortion was documented in 77.1% of patients, with 20.9% having undergone more than two abortions. No significant differences were observed in gravidity or abortion history. Preoperative hormone therapy was recorded in 239 of 339 patients (70.5%) in the focal adenomyosis group and 160 of 226 patients (70.8%) in the diffuse adenomyosis group, with no significant difference between groups (P = 0.149).

**Table 1:** Characteristics of patients with adenomyosis.

Variables	Categories	Results (n=685)
Age (years)		44.03 ± 6.62
	<35	78/685 (11.4)
	35-44	290/685 (42.3)
	45-54	300/685 (43.8)
	≥55	17/685 (2.5)
BMI^*1^ (kg/m2)		25.14 ± 3.72
	<18.5	14/685 (2)
	18.5-23.9	265/685 (38.7)
	24-27.9	278/685 (40.6)
	>=28	128/685 (18.7)
Pathological Type (n,%)	Focal adenomyosis	404/685 (59)
	Diffuse adenomyosis	281/685 (41)
Gravity(n,%)	0	28/664 (4.2)
	≤3	431/664 (64.9)
	4-5	183/664 (26.7)
	≥6	22/664 (3.2)
Parity(n,%)	0	57/664 (8.6)
	1-2	577/664 (86.9)
	>2	241/664 (4.5)
Abortion(n,%)	0	152/664 (22.9)
	1-2	373/664 (56.2)
	>2	139/664 (20.9)
Uterine Volume (mm^3^)^*2^		250.07 (162.36, 375.24)
Complaints (n,%)	menorrhagia	465/685 (67.9)
	irregular menstrual cycle	230/685 (33.6)
	Pelvic Pain/Dysmenorrhea	512/685 (74.7)
Asymptomatic(n,%)		41/685 (5.99)
History of Hormone Therapy(n,%)		399/565(70.62)
Surgical Modality(n,%)		
hysterectomy		491/685 (71.68)
uterine-sparing	Excision of adenomyosis	147/685 (21.46)
	Uterine Artery Embolization	4/685 (0.58)
	Hysteroscopy^*3^	43/685 (6.28)

^*1^ according to the Working Group on Obesity in China (WGOC) criteria.

^*2^ Uterine Volume (mm^3^)=π/6 *length*width*thickness (based on ultrasound measurement).

^*3^ Patients with ultrasound-diagnosed adenomyosis who underwent hysteroscopy due to endometrial lesions.

For continuous variables, data are presented as means ± SD or M(Q1-Q3); and for qualitative variables, the data are reported as number (percentage).

SD, standard deviation; M, Median; Q_1,_ 1st Quartile; Q_3_, 3st Quartile; BMI, body mass index.

**Table 2:** Demographic results of patients with diffuse and focal adenomyosis.

Variables	Fo-ADS (n=404)	Di-ADS (n=281)	P-Value
Age (years)	43.13 ± 6.55	45.33 ± 6.51	<0.001
BMI (kg/m2)	24.81 ± 3.62	25.62 ± 3.82	0.005
Gravidity (times)	2.92 ± 1.62	2.97 ± 1.65	0.973
Parity (times)	1.28 ± 0.73	1.41 ± 0.74	0.011
Abortion (times)	1.66 ± 1.41	1.56 ± 1.39	0.281
Uterine Volume (mm^3^)	248.86 (165.77,368.24)	254.73 (156.37,382.47)	0.946
Hemoglobin (g/L)	106.07 ± 23.91	102.47 ± 25.29	0.059
Fibrinogen (g/L)	2.74 ± 0.50	2.78 ± 0.54	0.348
D-dimer (mg/L)	0.09 (0.06, 0.14)	0.10 (0.06, 0.15)	0.185
SII^*1^	538.70 (380.76, 768.64)	554.78 (389.83,863.82)	0.444
CA125 (IU/ml)	80.7 (42.2,161.0)	52.7 (24.85,121.5)	<0.001
History of intrauterine surgery (n,%)	223/374 (59.6)	154/244 (63.1)	0.385
History of Hormone Therapy(n,%)	239/339 (70.50)	160/226 (70.8)	0.149
Pelvic Pain/Dysmenorrhea (n,%)	288/404 (71.3)	224/281 (79.7)	0.013
VAS score (n,%)			0.004
0 (no pain)	99/404 (24.5)	74/281 (26.3)	
1-3(mild)	147/404 (36.4)	71/281 (25.3)	
4-6(moderate)	97/404 (24)	69/281 (24.6)	
7-10(severe)	61/404 (15.1)	67/281 (23.8)	
moderate to severe pain	158/404 (39.1)	136/281 (48.4)	0.016
menorrhagia (n,%)	272/404 (67.3)	193/281 (68.7)	0.708
Irregular menstrual cycle (n,%)	120/404 (29.7)	110/281 (39.1)	0.010

^*1^ SII, Systemic Immune-Inflammation Index, platelet count × neutrophil count/.

lymphocyte count.

For continuous variables, data are presented as means ± SD or M(Q1-Q3); and for qualitative variables, the data are reported as number (percentage).

SD, standard deviation; M, Median; Q_1_, 1st Quartile; Q_3_, 3st Quartile; BMI, body mass index; VAS, visual analogue scale.

Regarding clinical symptoms, dysmenorrhea and pelvic pain were the most prevalent symptoms (74.7%), followed by menorrhagia (67.9%) and irregular menstrual cycles (33.6%); only 5.99% of patients were asymptomatic. Between-group comparisons showed that the Di-ADS group exhibited a higher prevalence of pain (79.7% vs. 71.3%, P = 0.013), with a substantially greater proportion experiencing moderate-to-severe pain (48.4% vs. 39.1%, P = 0.016). Irregular menstrual cycles were also more frequent in the Di-ADS group (39.1% vs. 29.7%, P = 0.010). However, there was no significant difference in the prevalence of menorrhagia. There was a marked contrast in serum CA125 levels between the two groups (52.7 vs. 80.7 IU/mL, P<0.001). Other parameters, including fibrinogen, hemoglobin, D-dimer, SII, uterine volume, and history of intrauterine surgery, did not differ statistically between subtypes. Surgery predominantly involved hysterectomy (71.68%), uterine-sparing approaches (21.46%) including adenomyosis excision, hysteroscopy for endometrial lesions (6.28%), and uterine artery embolization (UAE) for the management of acute heavy uterine bleeding (0.58%). The characteristics of the patients with diffuse and focal adenomyosis are summarized in **Table 2**.

Indications for operation included persistent menorrhagia, moderate–severe dysmenorrhea, abnormal endometrial or cervical pathology, endometriosis, ovarian neoplasm, and gynecologic cancer. Significant differences in surgical and comorbidity profiles were observed between the two groups (**Table 3**). While the overall incidence of adhesions was incomparable, the prevalence of recto-uterine pouch adhesion was higher in the Fo-ADS group (26.3% vs. 19.0%, P = 0.030). Endometriosis was also more frequent in Fo-ADS (29.7% vs. 17.1%, P<0.001). Consequently, the overall incidence of endometrial lesions was substantially greater in the Di-ADS group (49.5% vs. 35.6%, P<0.001). The significantly higher prevalence of endometrial lesions in the Di-ADS group was primarily driven by benign endometrial polyps (37.7% vs. 30.2%, P = 0.040) and atypical endometrial hyperplasia (6.76% vs. 1.24%, P<0.001). Conversely, the incidences of endometrial hyperplasia without atypia (2.49% vs. 2.48%, P = 0.990) and malignant endometrial cancer (2.5% vs. 1.7%, P = 0.572) did not show statistically significant differences between the diffuse and focal groups, although this may be partly attributed to the relatively small number of cases for these specific severe pathologies. No statistically significant differences were observed in the incidence of uterine leiomyomas (63.4% vs. 55.87%, P = 0.089), cervical intraepithelial neoplasia (5.0% vs. 4.6%, P = 0.989), benign ovarian cysts or tumors (19.3% vs. 22.4%, P = 0.371), or thyroid diseases (5.8% vs. 6.2%, P = 0.962). In addition, there were 4 cases of ovarian cancer and 2 cases of cervical cancer in the Fo-ADS group and 2 cases of cervical cancer in the Di-ADS group.

**Table 3:** Commodities of patients with focal and diffuse adenomyosis.

Variables (n,%)	Fo-ADS (n=404)	Di-ADS (n=281)	P-Value
Adhesion in surgery	225/392(57.4)	150/265(56.6)	0.840
Recto-uterine pouch adhesion	103/391 (26.3)	50/263 (19.0)	0.030
Endometriosis	120/404 (29.7)	48/281 (17.1)	<0.001
Uterine leiomyomas	256/404 (63.4)	157/281 (55.9)	0.089
Endometrial lesions^*1^	144/404 (35.6)	139/281 (49.5)	<0.001
Endometrial polyp	122/404 (30.2)	106/281 (37.7)	0.040
endometrial hyperplasia without atypia	10/404 (2.5)	7/281 (2.5)	0.990
Endometrial atypical hyperplasia	5/404 (1.2)	19/281 (6.8)	<0.001
Malignant endometrial cancer	7/404 (1.7)	7/281 (2.5)	0.572
CIN	20/404 (5.0)	13/281 (4.6)	0.989
Benign ovarian cyst/tumor	78/404 (19.3)	63/281 (22.4)	0.371
Thyroid Diseases	23/399 (5.8)	17/276 (6.2)	0.962

^*1^ include endometrial polyp, hyperplasia and cancer.

CIN, Cervical Intraepithelial Neoplasia.

The data are reported as number (percentage).

To identify predictors distinguishing diffuse from focal adenomyosis, multivariate logistic regression analysis was performed with focal adenomyosis as the reference group (**Table 4**). After adjusting for confounding variables, age, BMI, pain severity, and endometriosis emerged as factors associated with adenomyosis subtype. Advanced age (OR = 1.047, 95% CI: 1.021-1.075, P< 0.001), higher BMI (OR = 1.052, 95% CI: 1.007-1.099, P = 0.024), moderate-to-severe pain (OR = 1.436, 95% CI: 1.039-1.984, P = 0.028) were identified as significant risk factors for diffuse adenomyosis. Conversely, endometriosis was significantly more characteristic of the Fo-ADS group (OR = 0.459, 95% CI: 0.311-0.678, P<0.001).

**Table 4:** Risk factors of focal and diffuse adenomyosis.

Variables	Focal adenomyosis (versus Diffuse adenomyosis)		
	OR	95%CI	P
Age	1.047	1.021-1.075	0.000
BMI	1.052	1.007-1.099	0.024
Moderate to severe pain	1.436	1.039-1.984	0.028
Parity	1.122	0.894-1.409	0.319
Endometriosis	0.459	0.311-0.678	0.000

Multinomial logistic regression with focal adenomyosis as the reference group.

CI, confidence interval; OR, odds ratio.

**Figure 1:**
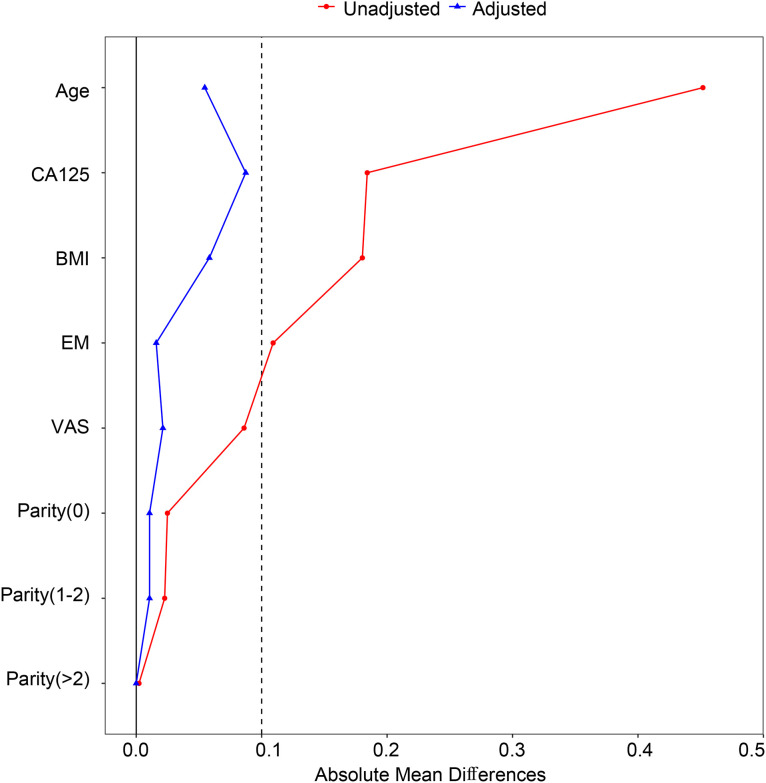
SMD plot showing the balance of covariates before and after PSM. This Love plot illustrates the absolute mean differences for baseline clinical characteristics between Fo-ADS and Di-ADS groups. The vertical dashed line indicates the SMD threshold of 0.1. Before Matching (Unadjusted, Red Circles), Significant imbalances were observed in several variables, particularly Age, CA125, and BMI, with SMD values well exceeding the threshold of 0.1. After Matching (Adjusted, Blue Triangles), Following 1:1 PSM, all covariates achieved optimal balance. BMI, body mass index; CA125, carbohydrate antigen 125; EM, endometriosis; VAS, visual analog scale for pain; PSM, propensity score matching.

To minimize confounding bias, 1:1 PSM was performed. After that, a total of 376 patients were successfully matched, with 188 cases in each group. Post-matching analysis demonstrated excellent balance between groups (**Table 5**). Quantitatively, SMDs for all covariates were reduced to <0.1, and no differences in baseline characteristics remained between the matched groups. (**Figure 1**, **Supplementary Figure 1**) To further control for residual confounding, multivariate conditional logistic regression analysis was performed (**Table 6**). Although baseline characteristics were balanced, age and BMI were included in the model to ensure doubly robust estimation of the treatment effect. Diffuse adenomyosis was independently associated with a significantly increased risk of endometrial lesions compared to focal adenomyosis (adjusted OR = 2.05, 95% CI: 1.28-3.27, P = 0.003).

**Table 5:** Baseline characteristics before and after propensity score matching.

Variable	Before PSM						After PSM					
	Total (n = 685)	1 (n = 404)	2 (n = 281)	Statistic	P	SMD	Total (n = 376)	1 (n = 188)	2 (n = 188)	Statistic	P	SMD
Age, Mean ± SD	44.03 ± 6.62	43.13 ± 6.55	45.33 ± 6.51	t=-4.335	<.001	0.338	45.16 ± 6.07	44.99 ± 6.10	45.34 ± 6.05	t=-0.552	0.582	0.057
BMI, Mean ± SD	25.14 ± 3.72	24.81 ± 3.62	25.62 ± 3.82	t=-2.806	0.005	0.211	25.51 ± 3.73	25.41 ± 3.67	25.62 ± 3.79	t=-0.569	0.570	0.058
CA125, M (Q_1_, Q_3_)	72.80 (34.62, 144.00)	80.70 (42.25, 159.50)	52.70 (25.00, 121.00)	Z=-3.947	<.001	-0.187	66.50 (31.48, 125.50)	75.05 (37.58, 141.75)	52.85 (24.93, 116.50)	Z=-2.691	0.007	-0.086
Endometriosis, n (%)				χ²=14.680	<.001					χ²=0.153	0.696	
no	514 (75.37)	281 (70.07)	233 (82.92)			0.341	303 (80.59)	150 (79.79)	153 (81.38)			0.041
yes	168 (24.63)	120 (29.93)	48 (17.08)			-0.341	73 (19.41)	38 (20.21)	35 (18.62)			-0.041
Parity, n (%)				χ²=0.529	0.768					χ²=0.155	0.925	
0	57 (8.58)	35 (8.97)	22 (8.03)			-0.035	28 (7.45)	15 (7.98)	13 (6.91)			-0.042
1-2	577 (86.9)	339 (86.92)	238 (86.86)			-0.002	332 (88.3)	165 (87.77)	167 (88.83)			0.034
>2	30 (4.52)	16 (4.10)	14 (5.11)			0.046	16 (4.26)	8 (4.26)	8 (4.26)			0.000
VAS, n (%)				χ²=5.838	0.016					χ²=0.170	0.680	
none or mild	391 (57.08)	246 (60.89)	145 (51.60)			-0.186	188 (50)	92 (48.94)	96 (51.06)			0.043
moderate to severe pain	294 (42.92)	158 (39.11)	136 (48.40)			0.186	188 (50)	96 (51.06)	92 (48.94)			-0.043

**Table 6:** Multivariate logistic regression after PSM.

Variables	β	S.E	Z	P	OR (95%CI)
Subtype					
Fo-ADS					1.00 (Reference)
Di-ADS	0.72	0.24	3.00	0.003	2.05 (1.28 - 3.27)
Age	-0.06	0.04	-1.52	0.129	0.94 (0.87 - 1.02)
BMI	-0.00	0.05	-0.05	0.959	1.00 (0.91 - 1.09)

CI, confidence interval; OR, odds ratio. Analyses were adjusted for age and BMI using conditional logistic regression stratified by matched pairs.

**Figure 2:**
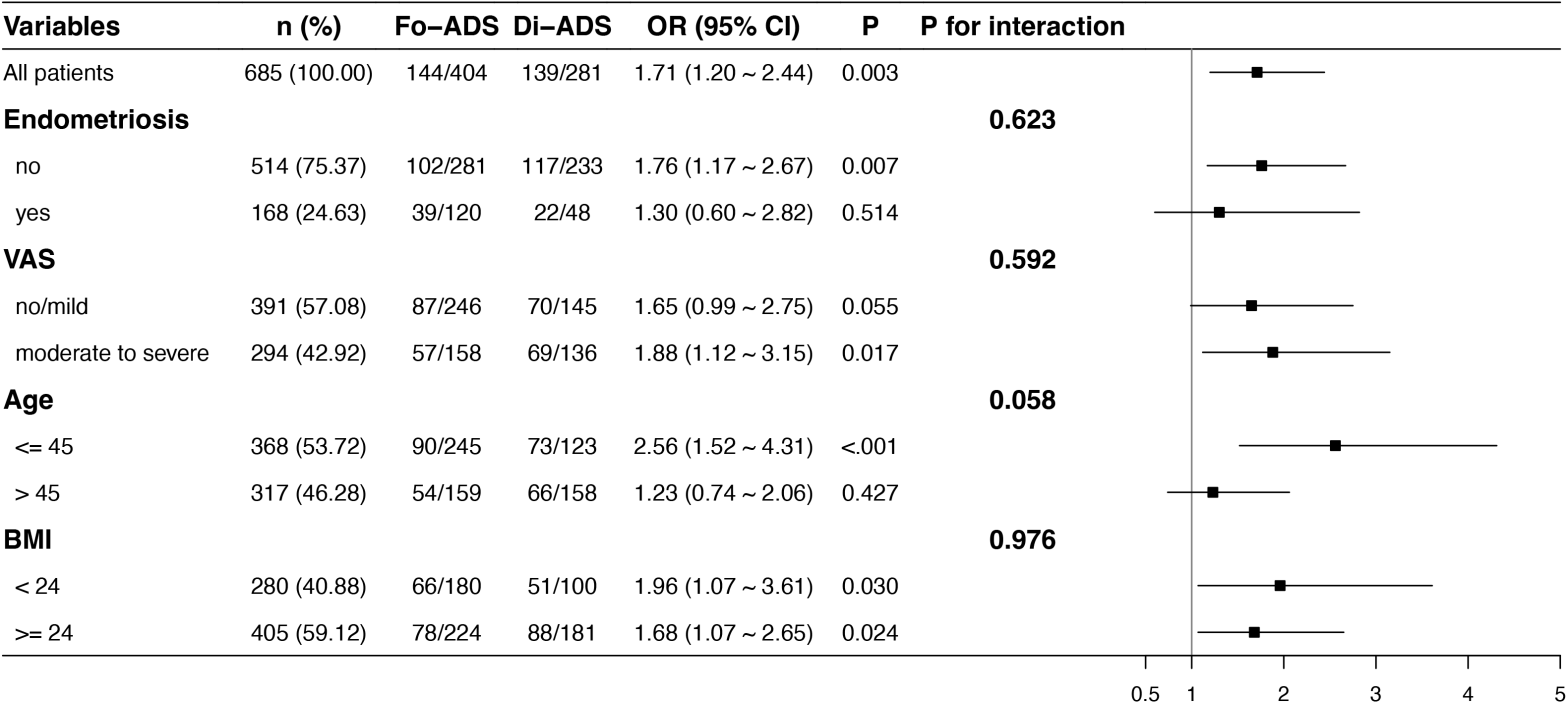
Subgroup analysis of the association between adenomyosis subtypes and the risk of endometrial lesions. The forest plot illustrates the risk of co-existing endometrial lesions in patients with Di-ADS compared to those with Fo-ADS across various clinical subgroups. The squares represent the odds ratios (ORs), and the horizontal lines represent the 95% confidence intervals (CIs). AM, adenomyosis; BMI, body mass index; VAS, visual analog scale for pain; EM, endometriosis; OR, odds ratio; CI, confidence interval. Adjusted by covariates including age, BMI, parity, and hormone therapy.

Subgroup analyses demonstrated that Di-ADS was consistently associated with an increased risk of endometrial lesions across all evaluated patient subgroups (aOR = 1.71, 95% CI: 1.20 ~ 2.44, P < 0.003). Interestingly, a marginal interaction was observed for age (P for interaction = 0.058), with a potentially stronger association observed in the younger subgroup (≤45 years) compared to the older subgroup (>45 years).(**Figure 2**) These age-stratified findings suggest that the impact of diffuse adenomyosis on endometrial lesion is notable in younger, premenopausal women, warranting closer clinical monitoring in this specific demographic.

## Discussion

Adenomyosis, defined by ectopic endometrial tissue within the myometrium, encompasses heterogeneous lesion distributions, including diffuse and focal forms. Building on this concept, recent literature supports the clinical and pathogenic dichotomy of adenomyosis that we observed in our cases (9, 18). Previous studies have documented the association between adenomyosis and endometrial lesions (17, 19); however, none have investigated the relationship between adenomyosis subtypes and endometrial lesions. To address this gap, our study used PSM and subgroup analysis to demonstrate that diffuse adenomyosis is associated with significantly higher rates of endometrial lesions, an effect particularly pronounced in women aged 45 years or younger. These findings underscore the need for careful evaluation and management of endometrial pathology in women with diffuse adenomyosis, especially in the premenopausal population.

In this retrospective study of 685 women, we comprehensively evaluated medical histories, ultrasound features, and intraoperative findings. Our results both confirmed and extended previous observations. We found that Di-ADS represents a phenotype associated with older age and higher BMI and is characterized by more severe dysmenorrhea compared with Fo-ADS. In contrast, Fo-ADS is characterized by elevated CA125 levels (80.7 vs. 52.7 IU/mL, P <0.001) and a higher prevalence of recto-uterine adhesions. These findings are highly consistent with the large-scale study by Li et al., which included 1,350 histologically confirmed cases and demonstrated that diffuse adenomyosis is associated with older age, higher gravidity, higher BMI, and severe dysmenorrhea. Li et al. proposed that diffuse adenomyosis represents a “progressive phenotype” primarily driven by the TIAR mechanism resulting from repeated mechanical or physiological disruptions of the EMI (9). Circumstantial evidence further suggests that chronic uterine peristaltic activity induces microtraumatization at the EMI near the fundo-cornual raphe, resulting in local estrogen production and self-perpetuation of the disease process (20). The association of Di-ADS with older age and higher parity supports the concept that diffuse adenomyosis represents a progressive stage of disease driven by cumulative tissue injury over time.

Conversely, the higher surgical complexity and CA125 levels in Fo-ADS are attributable to the adhesive disease caused by coexisting endometriosis. Li et al. found that focal and posterior uterine wall lesions exhibited a 3.24-fold increased risk of concurrent endometriosis and greater surgical complexity (9). This perfectly aligns with our observation, reinforcing the hypothesis that focal lesions share intrinsic pathobiological and inflammatory pathways with DIE. Gynecologists should be careful to dissect the adhesion of the recto-uterine pouch in patients with focal adenomyosis, particularly those with elevated CA125 levels. However, it should be noted that this elevation of serum CA125 is likely driven by the high comorbidity of endometriosis in focal cases rather than the adenomyosis lesion itself. Similarly, Chapron et al. demonstrated that focal adenomyosis located in the outer myometrium was observed more frequently in women with endometriosis and was significantly associated with the DIE phenotype, whereas diffuse adenomyosis showed no such correlation (21). In conjunction with existing evidence, our findings support the emerging paradigm that focal adenomyosis shares a common pathogenesis with pelvic endometriosis. This ‘outside-in’ mechanism, likely involving serosal-side infiltration of endometrial cells, is fundamentally distinct from the intrinsic origin of diffuse adenomyosis through invagination of the endometrial basalis (22).

The clinical distinction between these phenotypes is further supported by the work of Bourdon et al., who reported that focal adenomyosis was more common in young and nulliparous women and was associated with DIE, whereas diffuse adenomyosis was more often associated with heavy menstrual bleeding (18). However, we did not observe statistically significant differences in uterine volume or the incidence of menorrhagia between Di-ADS and Fo-ADS. This apparent discrepancy likely reflects the impact of preoperative medical management rather than true equivalence in the natural history of the disease. Many patients received gonadotropin-releasing hormone agonists (GnRH-a) or the levonorgestrel-releasing intrauterine system (LNG-IUS) to control symptoms prior to surgery. These interventions are known to reduce uterine volume and induce amenorrhea or hypomenorrhea, potentially masking baseline differences in uterine size and menstrual bleeding (23, 24). In addition, the distribution of preoperative hormonal therapy was similar between the two groups in our study. Consistently, no significant differences in hemoglobin levels or coagulation parameters were found between subtypes because patients with severe anemia typically underwent iron supplementation or transfusion to meet surgical safety criteria.

Our data also extend previous observations regarding the association between adenomyosis and endometrial pathology by demonstrating that this relationship is strongly subtype-dependent. In a large multicenter study of 1,455 hysterectomy specimens with adenomyosis, almost 15% had endometrial cancer, and endometrial polyps were among the most frequent coexisting pathologies (12). Yeh et al. demonstrated in a population-based cohort of 12,447 women that those with adenomyosis had a higher risk of endometrial cancer (HR = 2.19; 95% CI: 1.51–3.16) compared to adenomyosis-free women (19). This high concurrence reflects a profound pathophysiological continuum rather than mere coincidence, highlighted by Shiwali et al. who reported concurrent hyperplasia in 64.4% of patients with both adenomyosis and endometrial cancer (25). However, neither study stratified by adenomyosis phenotype. In our research, the Di-ADS group demonstrated significantly higher rates of endometrial lesions (49.5% vs. 35.6%, P<0.001). The higher prevalence of atypical hyperplasia in the diffuse subtype (6.8% vs. 1.2%, P< 0.001) underscores the importance of comprehensive endometrial evaluation in these patients. After PSM (188 matched pairs), Di-ADS maintained a significant independent association with endometrial lesions (aOR=2.05, 95% CI: 1.28–3.27, P = 0.003). This robust association suggests that the observed risk is not merely an artifact of baseline imbalances in age, BMI, or other potential confounders. These results are consistent with earlier work by Bergholt et al., who found that endometrial hyperplasia at hysterectomy was the variable significantly associated with adenomyosis (OR = 3.0; 95% CI: 1.2–8.3) (13), and with Indraccolo et al., who demonstrated significant associations between adenomyosis and both endometrial polyps (P = 0.013) and multiple endometrial polyps (P = 0.016), supporting a pathogenetic link between these conditions (26). Notably, while various endocrine and metabolic factors may influence endometrial pathology, our analysis demonstrated that preoperative hormonal interventions were comparably distributed between the focal and diffuse groups. Furthermore, key metabolic factors like BMI were balanced via PSM. These observations reinforce the hypothesis that the diffuse phenotype may possess an intrinsically distinct, hyperproliferative microenvironment that promotes endometrial dysregulation. However, we acknowledge that the composite “endometrial lesions” in this study encompass a spectrum of pathological subtypes. Although a lesion-specific analysis would been preferable, the relatively low frequency of events precluded robust multivariable modeling and increased the risk of statistical overfitting. Consequently, these findings should be regarded as an exploratory analysis of the association between adenomyosis subtypes and endometrial pathology, highlighting the need for larger prospective cohorts to clarify lesion-specific risks and develop refined predictive models.

Another important observation of this study is the age-dependent modification of the association between diffuse adenomyosis and endometrial lesions. This relationship was particularly pronounced in women aged ≤ 45 years (OR = 2.56, 95% CI: 1.52–4.31, P<0.001) but became attenuated and statistically non-significant in older women. Such divergence suggests that the pathogenic interplay between diffuse adenomyosis and the eutopic endometrium is most clinically relevant in premenopausal women. Mechanistically, the stronger effect size observed in younger women may reflect the relatively low baseline risk of endometrial pathology in this demographic, making the impact of adenomyosis more discernible. Conversely, in perimenopausal women, the rising risk associated with chronic estrogen exposure may diminish the relative contribution of adenomyosis to endometrial dysregulation.

From a clinical perspective, these findings provide a risk-adapted framework for the management of adenomyosis. For women ≤45 years, clinicians should consider a lower threshold for endometrial evaluation, including timely hysteroscopy or biopsy when clinically indicated. Notably, this vigilant approach may be warranted even in the absence of abnormal uterine bleeding.

Several mechanisms may elucidate the association between diffuse adenomyosis and endometrial lesions. Fundamentally, both entities are estrogen-dependent and are characterized by a local estrogen excess and progesterone resistance (27). In diffuse adenomyosis, the disruption of the myometrial structure amplifies hormonal alterations, creating a global hyperestrogenic microenvironment within the uterus. Particularly in premenopausal women, regular ovulatory cycles coupled with enhanced estrogen synthesis forge a highly proliferative state. Furthermore, estrogen induces epithelial-mesenchymal transition in endometrial epithelial cells, enhancing their migratory and invasive properties (28). Finally, the chronic inflammatory microenvironment inherent to adenomyosis may promote the pathophysiological transition from benign proliferation to potential malignancy (29). However, it remains unclear whether diffuse adenomyosis preceded endometrial lesions or whether shared underlying pathophysiological factors contributed to both conditions.

Several limitations of this study must be noticed when interpreting these findings. First, with respect to lesion classification, we did not stratify adenomyosis.

by anterior versus posterior wall involvement, because we are focusing on the effects of different subtypes. Second, a subset of patients relied on ultrasonographic criteria while the majority of diagnoses were histologically confirmed. Although transvaginal ultrasound has high sensitivity (72–82%) and specificity (81–85%) for adenomyosis diagnosis, the lack of histological confirmation in these specific cases remains potential bias (30). Additionally, this retrospective study mainly enrolled patients who underwent hysterectomy and the cases tended to be older. As a result, the population was enriched for patients with surgical indications and may represent a more severe disease spectrum, introducing potential spectrum bias. Therefore, the prevalence of endometrial lesions observed in this study should not be generalized to all patients with adenomyosis, especially those managed conservatively. Nevertheless, this design strengthen the internal validity of the observed association. Finally, the relatively small number of severe endometrial pathologies precluded the development of a robust predictive model for specific lesion in the present study to ensure stable estimation and minimize the risk of overfitting. In the future, large-scale cohort studies are warranted to accumulate sufficient cases, which would allow the internal and external validation of comprehensive predictive models and more refined risk stratification systems.

## Conclusion

In summary, our findings suggest that focal and diffuse adenomyosis represent distinct clinical phenotypes with divergent comorbidity profiles. Focal adenomyosis was more frequently accompanied by pelvic adhesions and coexisting endometriosis, underscoring the need for careful surgical planning when these conditions are present. Diffuse adenomyosis was associated with a higher prevalence of endometrial lesions in surgically treated, pathologically confirmed cases particularly among women ≤45 years. These observations highlight an association between diffuse adenomyosis and endometrial pathology, but the temporal and causal relationship remains unclear. Future prospective studies incorporating molecular profiling and longitudinal follow-up are needed to validate these findings and further elucidate the underlying mechanisms.

## Data Availability

The raw data supporting the conclusions of this article will be made available by the authors, without undue reservation.

## Supplementary material

Supplementary Figure 1 Distribution of propensity scores before and after matching. The density plots display the distribution of propensity scores for patients in the Fo-ADS (Treatment 0, pink) and Di-ADS (Treatment 1, cyan) groups. Unadjusted Sample (Left), Prior to matching, a significant discrepancy in the distribution of propensity scores was observed between the two groups. Adjusted Sample (Right): After 1:1 PSM, the distributions of the two groups show a high degree of overlap. “Distance” on the x-axis represents the estimated propensity score.

Supplementary Figure 2 Representative images of paired ultrasound and histopathology for adenomyosis subtypes. **(A)** Transvaginal Ultrasound of focal adenomyosis: A localized, heterogeneous hypoechoic lesion is observed within myometrium (star); The arrow points to the endometrium. **(B)** Histopathology (H&E, 20x) of focal adenomyosis: The corresponding surgical specimen shows a circumscribed area of endometrial glands and stroma embedded within the myometrium. **(C)** Transvaginal Ultrasound of diffuse adenomyosis: The uterus exhibits global enlargement with asymmetrical thickening of the posterior wall; The arrow points to the endometrium. **(D)** Histopathology (H&E, 20x) of diffuse adenomyosis: Wide-scale, irregular infiltration of endometrial tissue into the myometrium is observed across multiple sections.
