# Supplementary figures and images for: Association between adenomyosis subtypes and concurrent endometrial lesions: a propensity score-matched retrospective study
Source: Front Endocrinol (Lausanne). 2026 May 29;17:1842535. doi: 10.3389/fendo.2026.1842535 (PMC13261174; doi:10.3389/fendo.2026.1842535)

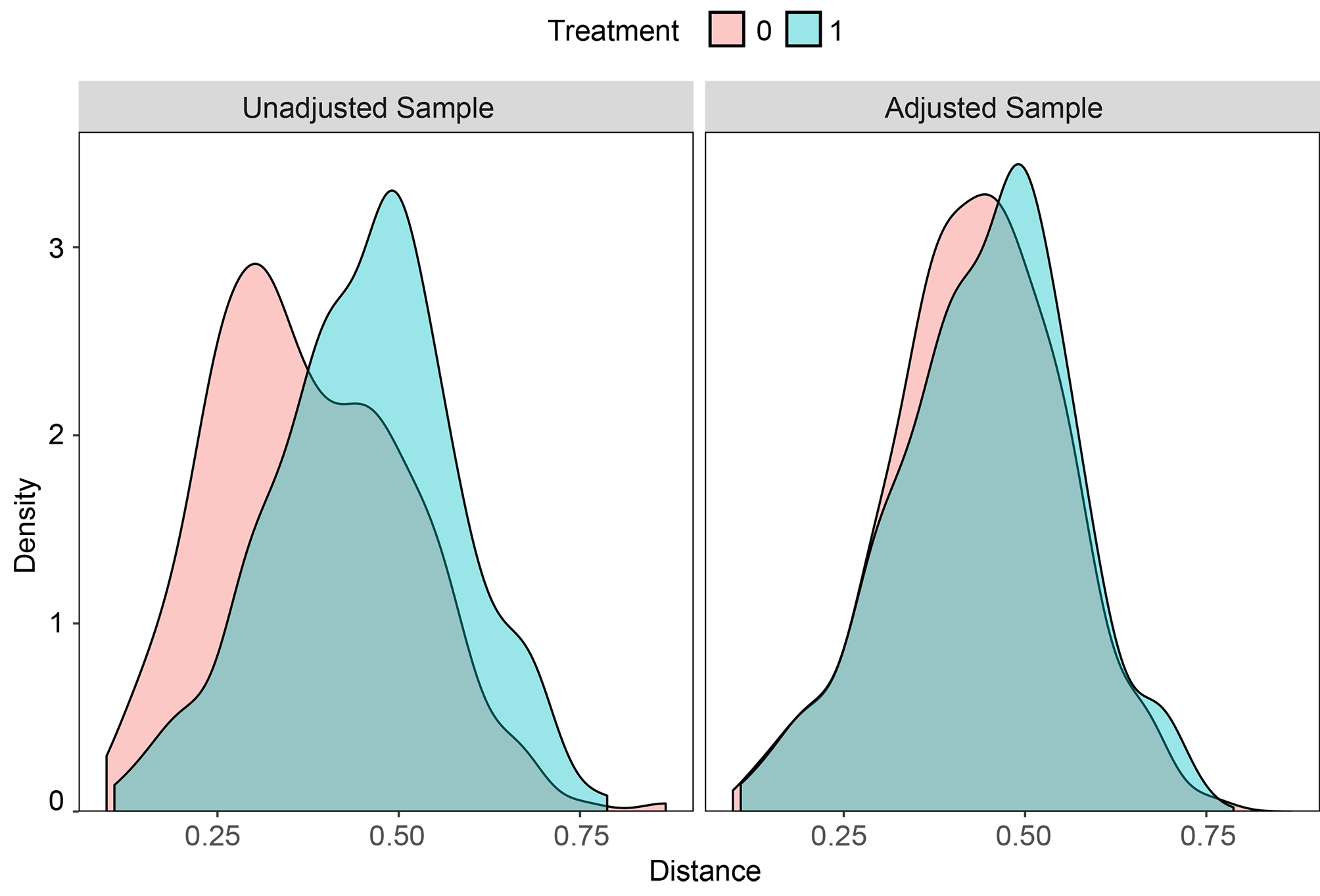

Supplement: Supplementary Figure 1 — Distribution of propensity scores before and after matching. The density plots display the distribution of propensity scores for patients in the Fo-ADS (Treatment 0, pink) and Di-ADS (Treatment 1, cyan) groups. Unadjusted Sample (Left), Prior to matching, a significant discrepancy in the distribution of propensity scores was observed between the two groups. Adjusted Sample (Right): After 1:1 PSM, the distributions of the two groups show a high degree of overlap. “Distance” on the x-axis represents the estimated propensity score. [file Image1.tif]

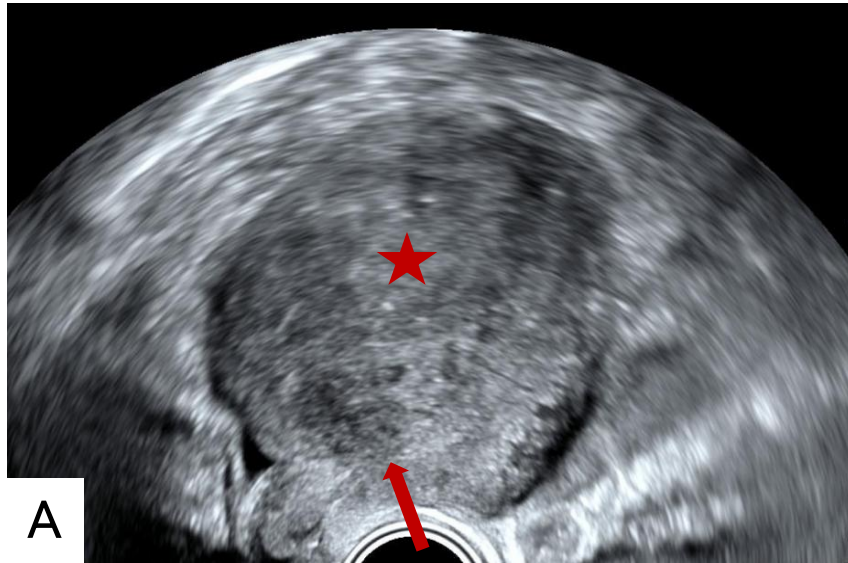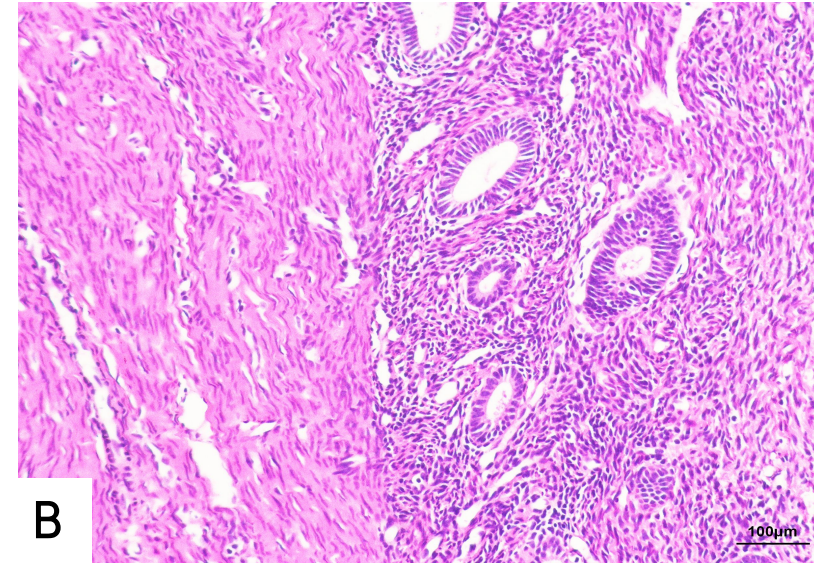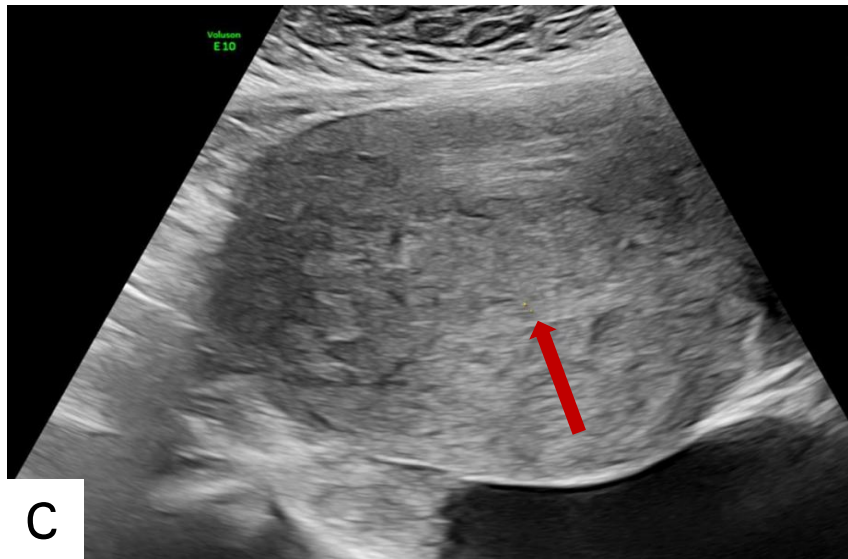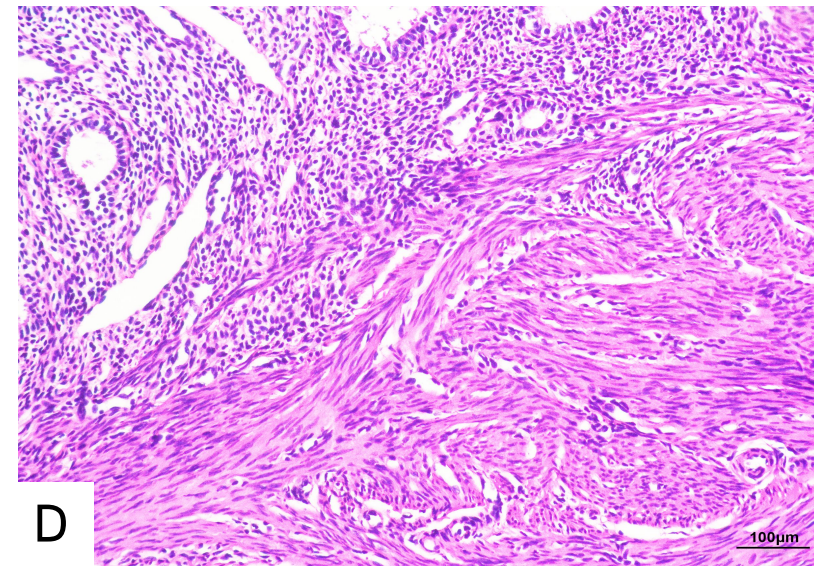

Supplement: Supplementary Figure 2 — Representative images of paired ultrasound and histopathology for adenomyosis subtypes. (A) Transvaginal Ultrasound of focal adenomyosis: A localized, heterogeneous hypoechoic lesion is observed within myometrium (star); The arrow points to the endometrium. (B) Histopathology (H&E, 20x) of focal adenomyosis: The corresponding surgical specimen shows a circumscribed area of endometrial glands and stroma embedded within the myometrium. (C) Transvaginal Ultrasound of diffuse adenomyosis: The uterus exhibits global enlargement with asymmetrical thickening of the posterior wall; The arrow points to the endometrium. (D) Histopathology (H&E, 20x) of diffuse adenomyosis: Wide-scale, irregular infiltration of endometrial tissue into the myometrium is observed across multiple sections. [file DataSheet1.pdf]
